# Les méningiomes intracrâniens opérés au CHU Sylvanus Olympio de Lomé: aspects anesthésiologiques et complications à propos de 21 cas

**DOI:** 10.11604/pamj.2017.28.42.11451

**Published:** 2017-09-15

**Authors:** Pilakimwé Egbohou, Tabana Mouzou, Kadanga Beketi, Essossinam Kpelao, Abdel Kader Moumouni, Hamza Doles Sama, Sarakawabalo Assénouwé, Gnimdou Akala-Yoba, Kadjika Tomta

**Affiliations:** 1 Service d’Anesthésie Réanimation du CHU Sylvanus Olympio de Lomé, Togo; 2 Service de Neurochirurgie du CHU Sylvanus Olympio de Lomé, Togo

**Keywords:** Méningiomes intracrâniens, anesthésie, complications, CHU Togo, Intracranial meningiomas, anesthesia, complications, Sylvanus Olympio University Hospital Center, Togo

## Abstract

L’objectif était de relever les aspects anesthésiologiques et les complications périopératoires des méningiomes intracrâniens opérés au CHU Sylvanus Olympio de Lomé (CHU SO). Il s’agissait d’une étude rétrospective sur dossiers de patients opérés de méningiomes intracrâniens durant la période de Décembre 2010 à décembre 2015 (5 ans) au CHU SO. 21 (45,6%) méningiomes recensés sur 46 tumeurs cérébrales opérées. Age moyen: 49 ± 20 ans; prédominance masculine (52,4%), ratio M/F: 1,1. Classification ASA: ASA II: 16 patients, ASA III: 4 patients, et ASA IV: 1 patient. L’anesthésie a été intraveineuse totale avec du Propofol (100%) comme hypnotique et du fentanyl (76,2%) comme morphinique le plus disponible. Complications peropératoires: saignement, pertes sanguines moyennes: 1750 ± 584 ml; hypotension avec Pression artérielle moyenne (PAM) < 60 mmHg: 10 (47,6%) patients; choc hémorragique 2 (9,5%) patients, arrêt cardiocirculatoire: 01(4,7%) patient réanimé avec succès. Complications postopératoires: convulsions 5 (23,8%) cas, hyperthermie 4 (19%) cas, choc hémorragique 2(9,5%) cas, décès: 2 (9,5%) cas. La morbidité et la mortalité périopératoire de la chirurgie du méningiome intracrânien au CHU SO de Lomé reste élevée. L’amélioration du plateau technique et une consultation précoce permettrait de réduire ces complications.

## Abstract (en)

This study aimed to point to both the anesthesiological aspects and the perioperative complications of intracranial meningiomas operated at the Sylvanus Olympio University Hospital Center, Lomé. We conducted a retrospective study by reviewing the medical records of patients with intracranial meningiomas undergoing surgery over the period December 2010-December 2015 (5 years) at the Sylvanus Olympio University Hospital Center, Lomé. Out of 46 patients operated fo brain tumors, 21 (45.6%) had meningioma. The average age was 49 ±20 years, with a male predominance (52.4%) and a sex ratio (M/F) of 1.1. Patients were classified according to ASA classification: 16 patients were classified as ASA II, 4 patients as ASA III and 1 patient as ASA IV. Patients underwent total intravenous anesthesia using hypnotic agent such as propofol (100%) and fentanyl (76.2%), which was the most available opioid. Perioperative complications were: bleeding, (mean blood loss: 1750 ±584 ml), hypotension (mean arterial pressure (MAP) < 60 mmHg) in 10 (47.6%) patients; hemorrhagic shock in 2 (9.5%) patients, cardiovascular arrest: 01 (4.7%) patient successfully resuscitated. Postoperative complications were: convulsions in 5 (23.8%) cases, hyperthermia in 4 (19%) cases, hemorrhagic shock in 2 (9.5%) cases, death in 2 (9.5%) cases. Perioperative morbidity and mortality associated with intracranial meningioma surgery at the Sylvanus Olympio University Hospital Center, Lomé remains high. Improvement of technical equipment and early consultation should reduce these complications.

## Introduction

Les méningiomes intracrâniens sont des tumeurs cérébrales, extra parenchymateuses habituellement bénignes développées aux dépens des cellules arachnoïdiennes [1]. La chirurgie du méningiome est une chirurgie majeure, hémorragique et de longue durée. L’anesthésie pour cette chirurgie impose de respecter de nombreux impératifs dont préserver l’autorégulation cérébrale et la réactivité au CO_2_, optimiser la compliance du cerveau pour diminuer la pression sous écarteurs, protéger le cerveau des agressions cérébrales secondaires d’origine systémique (ACSOS) [2]. La prise en charge anesthésiologique des méningiomes intracrâniens au Togo a déjà fait l’objet d’étude [3]. La présente étude dont l’objectif était de relever les aspects anesthésiologiques, et d’évaluer la morbidité et la mortalité périopératoire des patients opérés de méningiomes intracrâniens au CHU Sylvanus Olympio de Lomé se justifie par une plus longue période (5 ans) et une plus grande série de cas.

## Méthodes

Le service de neurochirurgie a servi de cadre à cette étude. Les interventions y sont réalisées par 3 neurochirurgiens et l’anesthésie est assurée par une équipe constituée d’un médecin anesthésiste et de 2 techniciens supérieurs d’anesthésie et réanimation. Il s’est agi d’une étude rétrospective sur dossiers de patients de décembre 2010 à décembre 2015 soit une période de 5 ans. Tous les dossiers de patients ayant bénéficié d’une anesthésie pour exérèse de méningiome intracrânien (avec confirmation histologique du diagnostic) ont été inclus dans notre étude. Une fiche d’enquête a servi au recueil des données. Les données démographiques, cliniques, chirurgicales, anesthésiologiques, les complications et les décès périopératoires ont été étudiés. Les moyens de monitorage disponibles étaient un moniteur de surveillance multiparamétrique avec une pression artérielle non invasive, une oxymétrie pulsée, un tracé ECG 5 dérivations, et une capnographie intégrée au ventilateur d’anesthésie. Deux protocoles anesthésiques avaient été utilisés. Le premier incluait le propofol, le fentanyl, le pancurium à l’induction, en entretien du propofol en perfusion continue à la séringue électrique, et du fentanyl en boli itératifs. Le second incluait le propofol, le sufentanil, le pancuronium à l’induction, et en entretien du propofol en continu à la séringue électrique et des boli de sufentanil. Indépendamment du protocole une benzodiazépine, le diazépam ou le midazolam était faite à l’induction, et la succinylcholine était disponible pour les intubations prévues difficiles. Le mannitol 20% (0,5g/kg de poids) était fait pour la détente cérébrale avant l’ouverture du volet, répéter au besoin à la demande du chirurgien. L’antibioprophylaxie était faite à base de Ceftriaxone 2g avant l’induction, répétée à 1g après 4h en peropératoire, et poursuivie pendant 3 jours à la dose de 2g/jour. La classification ASA de l’American Society of Anesthesiologist revue par Dripps RD, a été utilisée pour évaluer l’état clinique des patients à opérer [4]. L’exérèse tumorale réalisée basée sur les constations peropératoires du chirurgien a été décrite selon la classification de Donald Simpson [5]. Le saignement a été estimé en additionnant les pertes compensées (quantité de culots globulaires (CG) transfusés: 1 CG = 250 ml à 70% d’hématocrite (Ht) soit environ 500 ml à 35% Ht), aux pertes non compensées: Volume sanguin total x (Ht initial (J-1) – Ht final (J3) à 100% d’Ht à ramener à 35% Ht), Volume Sanguin Total = poids x 70 pour l’homme ou poids x 65 pour la femme en ml. Le dépouillement a été manuel et les données traitées par Epi info 3.5.

## Résultats

Sur 46 tumeurs cérébrales opérées, 21(45,6%) étaient des méningiomes intracrâniens. L’âge moyen des patients opérés était de 49 ±20 ans avec des extrêmes de 7 ans et 78 ans. Onze patients (52,4%) étaient de sexe masculin et le ratio M/F de 1,1. Dix-huit méningiomes étaient de la convexité dont 6 frontales, 4 fronto-pariétales, 5 temporales, 3 parasagittales. Trois méningiomes étaient localisées à la base dont un à la fosse postérieure et 2 sphénoïdales. La taille moyenne du grand axe des tumeurs était de 5,8 ± 1,4 cm. Un œdème péritumoral, un effet de masse et un engagement cérébral ont été retrouvé respectivement chez 15, 14, et 4 patients. Trois patients ont bénéficiés d’un degré d’exérèse chirurgicale de SIMPSON de grade I, 13 patients de grade II, 3 de grade III, et 2 de grade IV. Cinq patients avaient une anémie (Taux d’hémoglobine < 12 g/dl), 5 avaient une HTA traitée, 3 un diabète et 2 une cardiopathie ischémique. Seize patients étaient classés ASA II, 5 patients ASA III et 1 patient ASAIV. Le Tableau 1 donne le détail des données cliniques des patients opérés. Tous les patients avaient reçu du méthyl prednisolone, 10 patients avaient reçu du Valproate de Sodium en préopératoire. En peropératoire, l’anesthésie à été intraveineuse totale. Le Propofol a été le narcotique utilisé à l’induction et en perfusion au pousse-séringue électrique pour l’entretien de l’anesthésie. Parmi les morphiniques, le fentanyl (76,2%) était plus disponible que le sufentanil utilisé chez 5(23,8%) patients. Les curares disponibles étaient le pancuronium (100%) et la succinylcholine (4,7%) pour les patients à intubation difficile. Le mannitol 20% a été utilisé pour la détente cérébrale chez tous les patients.

**Tableau 1:** Caractéristiques cliniques des patients

	Effectifs	Pourcentages (%)
**Antécédents**		
Alcool	6	28,5
Anémie	5	23,8
HTA	5	23,8
Diabète	3	14,3
Cardiopathie ischémique	2	9,5
Tabac	2	9,5
Asthme	1	4,7
**Score de Glasgow**		
4 - 8	1	4,7
9 - 12	4	19
13 – 15	16	76,2
Œdème cérébral	15	71,4
Effet de masse important	14	66,7
Engagement cérébral	4	19
**Classes ASA**		
II	16	76,2
III	4	19
IV	1	4,7

Les complications peropératoires ont consisté essentiellement en saignement, les pertes sanguines moyennes estimées à: 1750 ±584 ml, engendrant des hypotensions avec PAM < 60 mmHg: 10 (47,6%) patients , des chocs hémorragiques: 2 (9,5%) patients et 01(4,7%) arrêt cardiocirculatoire réanimé avec succès. La correction de l’hypovolémie induite par le saignement a été faite par le remplissage avec du sérum salé 0,9% et de la gélofusine, mais aussi par des transfusions homologues. Il y a eu un recours aux amines vasopressives pour les cas réfractaires au remplissage (chocs hémorragiques) essentiellement de la noradrénaline, et de l’adrénaline pour la réanimation de l’arrêt cardiaque; le Tableau 2 donne le détail des médicaments utilisés durant l’intervention chirurgicale. Dix huits (85,7%) patients ont été transfusés. La quantité moyenne de culots globulaires (CG) transfusés était de 1200 ± 320 ml. Dix patients avaient en plus reçu en moyenne 1200 ml (3 unités adultes) de Plasma Frais Congélé (PFC). La durée moyenne des interventions était de 6,4 ±1,7 heures. Dix neuf patients (90,5%) ont été réveillés sur table et 2 sédatés en réanimation, du fait de la persistance du choc hémorragique. Les complications postopératoires ont été dominées par les convulsions 5 (23,8%), les hyperthermies 4 (19%), les états de choc hémorragique 2 (9,5%). Deux (9,5%) décès ont été enregistré dans le postopératoire en réanimation par choc hémorragique à J1 pour le premier, et sepsis sévère à J5 pour le second. Les résumés des complications périopératoires et des caractéristiques des patients décédés sont données par les Tableau 3 et Tableau 4.

**Tableau 2:** Médicaments utilisés en peropératoire

	Effectif	Pourcentage (%)
**Narcotiques**		
Propofol	21	100
Midazolam	6	28,5
Diazépam	15	71,4
**Morphiniques**		
Fentanyl	16	76,2
Sufentanil	5	23,8
**Curares**		
Pancuronium	21	100
Succinylcholine	1	4,7
**Autres medicaments**		
SS 0,9%	21	100
Mannitol 20%	21	100
Solumédrol	21	100
Ceftriaxone	21	100
Ephedrine	21	100
Gélofusine	21	100
Phénobarbital	8	38,1
Noradrénaline	2	9,5
Adrénaline	1	4,7

**Tableau 3:** Complications périopératoires

	Effectifs	Pourcentages (%)
**Complications per opératoires**		
Saignement > 1500 ml	18	85,7
Hypotension (PAM^+^< 60 mmHg)	10	47,6
Choc hémorragique	2	9,5
Arrêt cardiaque	1	4 ,7
**Complications postopératoires**		
Choc hémorragique	2	9,5
Convulsions	5	23,8
Cépahalées	2	9,5
Agitations	1	4,7
Aphasie	1	4,7
Hyperthermie	4	19
**Décès**	**2**	**9,5**

+ PAM: pression

**Tableau 4:** Profil des patients décédés

	Patient 1	Patient 2
Age (années)	66	73
Sexe	Masculin	Féminin
Antécédents	HTA	HTA, diabète, asthme
Score de Glasgow	12	13
ASA	II	III
Siège du méningiome	Frontal droit	Frontal gauche
Taille (cm)	5	6,5
œdème cérébral	Oui	Oui
Effet de masse	Important	Important
Engagement	Oui	Non
Exérèse(Simpson)	II	IV
Durée d’intervention	8h22	6h50
Saignement peropératoire	2,1 litres	3 litres
Complication postopératoire	Infection,	Hypotension, hyperthermie
Causes immédiates de décès	Sepsis sévère	Choc hémorragique
Délai postopératoire	J5	J1

## Discussion

Le caractère rétrospectif de l’étude et la petite taille de notre échantillon ont constitués des limites à cette étude. Nous avons recensé 21 méningiomes sur 46 tumeurs intracrâniennes opérées dans le service de neurochirurgie durant les 5 années de cette étude soit 45,6% de l’ensemble des tumeurs cérébrales opérées. En Côte d’Ivoire, en 2008, N’dri et coll trouvaient 114 cas de méningiomes sur 10 ans [6]. Badiane à Dakar en 1999, trouvait 18,7% de méningiomes sur 253 patients opérés pour tumeurs cérébrales [7]. En 2015 Thiam au Sénégal dans le même service réhabilité, rapportait 78 cas (18,22%) sur 5 ans [8]. Ibebuike et coll rapportent une incidence de 31,8% en 2013 en Afrique du Sud [9]. Aux Etats Unis des auteurs rapportent des taux de 23% de l’ensemble des tumeurs primitives du cerveau [10]. La petite taille de notre échantillon s’expliquerait par le caractère naissant de la neurochirurgie dans notre pays, la non référence des patients devant des symptômes d’alarme, mais aussi par les difficultés (financières) d’accès aux explorations d’imagerie indispensables au diagnostic. Dans notre étude, nous avons retrouvé une prédominance masculine (52,4%). La prédominance féminine est classique dans la littérature et aurait des justifications hormonales [7, 8, 11–13]. Un échantillon plus grand serait important pour juger de la réelle prédominance sexuelle dans notre pays. La moyenne d’âge de 49 ± 20 ans retrouvée dans notre étude concorde avec celles habituellement rencontrées, autour de la cinquantaine [7, 8, 11, 12].

Tous les patients de notre série ont bénéficié d’une anesthésie générale intraveineuse totale avec intubation trachéale et ventilation mécanique assistée. Dans notre étude le propofol a été l’hypnotique utilisé et le fentanyl (76,2%) le morphinique le plus utilisé parce que le plus disponible. L’analgésie produite par le fentanyl ou le sufentanyl est puissante mais le rémifentanyl est recommandé car ne s’accumulant pas et permettant un réveil rapide des patients. Le réveil rapide dans ce type de chirurgie est important pour une évaluation rapide de l’état neurologique du patient en vue d’un diagnostic précoce des complications [2]. Dans notre étude 19 (90,5%) patients ont été réveillé précocement sur table et aucune reprise opératoire précoce du fait de complications neurologiques n’a été faite. L’utilisation systématique du mannitol dans notre étude se justifie par la nécessité d’une détente cérébrale. Elle répond au concept d’écarteurs chimiques par amélioration de la compliance cérébrale essentiellement par le biais d’une réduction de la teneur en eau du parenchyme cérébral par création d’un gradient osmotique. D’autres solutés hypertoniques tels le sérum salé hypertonique à 7,5% sont également indiqués [14, 15].

La détection des complications peropératoires qui constituent des ACSOS a été rendu possible grâce au monitorage de la pression artérielle non invasive (PANI), de la saturation pulsée en oxygène (SPO2), de l’ECG 5 dérivations, du CO2 expiré (ETCO2) par un capnographe. En cours d’exérèse, 10 (47,5%) patients ont présenté une chute de la pression artérielle moyenne (PAM) en dessous de 60 mmHg. L’hypotension est un ACSOS grave qui ne doit souffrir d’aucun délai de prise en charge; elle réduit la perfusion cérébrale avec un risque d’ischémie cérébrale. Il est recommandé une PAM = 80 mmHg pour une pression de perfusion cérébrale (PPC) suffisante = 60 mmHg afin d’éviter une ischémie cérébrale [2, 16–18]. La diminution de la PAM a été le fait d’un saignement important. Les pertes sanguines moyennes dans notre étude étaient de 1750 ± 584 ml; 18 (85,7%) patients ont été transfusés avec une quantité moyenne de 1200 ± 320 ml de culots globulaires. Dans notre étude, l’hémostase chirurgicale a été réalisée avec un bistouri électrique bipolaire et le recours à du gaze résorbable d’oxycellulose. Les anses électriques n’étaient pas disponibles donc non utilisées. Kenza L. a recensé sur 105 patients opérés de méningiomes intracrâniens entre 2000 à 2009 au Maroc, 46,19% de saignement excessif et 37,1% de ses patients ont été transfusés [19]; Toumi, a trouvé dans son étude sur 100 patients de 2002 à 2004 une perte sanguine moyenne de 1,57 ± 0,73 litre, l’incidence de la transfusion dans son étude était de 52%. Elle retrouve comme facteurs prédictifs de saignement, la taille scanographique de la tumeur, la durée d’intervention, le délai entre les symptômes et le diagnostic [20]. La durée moyenne des interventions était de 6,4 ± 1,7h. Des durées moins élevées ont été retrouvées par Kenza (4h45 ±1h36 min) et Toumi (6,023h ± 1,4h) [19, 20]. Cette durée d’intervention plus longue dans notre étude s’expliquait par les conditions techniques difficiles de réalisation de cette chirurgie au CHU SO de Lomé: plateau technique insuffisant, ressources humaines limitées seulement 1 chirurgien durant les 2 premières années de l’étude.

Nous avons enregistré dans notre étude deux décès soit 9,5% des cas. Les causes immédiates des décès retrouvées dans notre étude étaient hémodynamiques par choc hémorragique au premier jour, pour le premier et par sepsis sévère pour le second au 5^ème^ jour. Les deux patients décédés étaient âgés de plus de 65 ans et présentaient des comorbidités: HTA, diabète, asthme. N’dri trouvait 12,63% en Côte d’Ivoire, et Thiam au Sénégal trouvait 16% (8 patients sur 50 dossiers documentés) avec des causes hémodynamiques, infectieuses, respiratoires et chirurgicales [6, 8]. Kenza et Toumi au Maroc trouvaient des taux plus bas de 8,6%, et 4% au Maroc [19, 20].

## Conclusion

Les impératifs communs à la prise en charge anesthésiques des tumeurs cérébrales restent: la préservation de l’autorégulation cérébrale et de la réactivité au CO_2_, l’optimisation de la compliance du cerveau, la protection contre les ACSOS. A ces impératifs il faudra ajouter pour la prise en charge des méningiomes intracrâniens, la gestion des saignements importants, et de longues durées d’intervention. Les complications infectieuses sont possibles comme dans notre étude et amènent à repenser l’asepsie au bloc opératoire et à réadapter l’antibioprophylaxie.

### Etat des connaissances actuelles sur le sujet

- La chirurgie du méningiome est une chirurgie longue, hémorragique qui impose à l’anesthésiste de respecter certaines contraintes à savoir prendre en charge l’hypertension intracrânienne générée par la tumeur;
- Eviter la survenue d’agressions cérébrales secondaires d’origine systémiques telles l’hypotension et l’hypoxie responsables d’ischémie cérébrale.

### Contribution de notre étude à la connaissance

- La morbi-mortalité dans la prise en charge périopératoire des méningiomes intracrâniens au CHU SO de Lomé est liées essentiellement à des complications hémorragiques et infectieuses.

## Conflits d’intérêts

Les auteurs ne déclarent aucun conflit d'intérêts.
